# Steatocystoma multiplex suppurativa: case report of a rare condition*

**DOI:** 10.1590/abd1806-4841.20164539

**Published:** 2016

**Authors:** Cândida Naira Lima e Lima Santana, Daniele do Nascimento Pereira, Alice Paixão Lisboa, Juliana Martins Leal, Daniel Lago Obadia, Roberto Souto da Silva

**Affiliations:** 1 Universidade do Estado do Rio de Janeiro (UERJ) – Rio de Janeiro (RJ), Brazil; 2 Hospital Central do Exército (HCE) – Rio de Janeiro (RJ), Brazil; 3 Universidade do Grande Rio (Unigranrio) – Rio de Janeiro (RJ), Brazil

**Keywords:** Steatocystoma Multiplex, Hidradenitis Suppurativa, Suppuration

## Abstract

Steatocystoma multiplex is a rare genetic disorder characterized by the presence of hamartomatous malformations at the junction of the pilosebaceous duct. It consists of encapsulated cystic lesions in the dermis, with adjacent sebaceous gland. When associated with inflammation, resembling hidradenitis, it is called steatocystoma multiplex suppurativa, a condition rarely reported. This is the first case of steatocystoma multiplex suppurativa reported in the Brazilian literature. Female patient, 23 years old, with papular and nodular cystic lesions that started in the armpits and groin, later spreading to the trunk, lower limbs, anticubital fossa, face and scalp. The presence of papular-nodular lesions associated with disseminated hidradenitis-like lesions in flexural areas and the histopathological diagnosis of steatocystoma defined the diagnosis of steatocystoma multiplex suppurativa.

## INTRODUCTION

Steatocystoma multiplex (SM) is a rare genetic disorder.^1,2^ It is characterized by multiple dermal cysts of varying sizes, which present in areas where the pilosebaceous unit is well developed, predominantly in armpits, trunk and proximal ends.^3-6^ In women, SM presents with greater frequency in the inguinal region and in men it is usually arranged in a diamond-shape distribution in the trunk. Rarely, it is found on the scalp and face.^1-3,7^ Lesions vary from normochromic to yellow, are movable, with slow growth and liquid content most of the times.^2,4^

SM appears in adolescence or early adulthood, with no predilection for sex.^1,2^ Most cases have autosomal dominant feature, but it can also occur sporadically.^5,6,8^

Although it is a benign condition and most of the lesions are asymptomatic, there is an inflammatory variant called SM suppurativa.^4,6^ In this case, there is spontaneous rupture of the cysts, suppuration and malodorous drainage, especially if secondarily colonized by bacteria.^2,6,9^ Abscesses can also occur. These findings resemble to lesions of acne conglobata or hidradenitis suppurativa.^9^ The lesions can progress to scars, causing much discomfort to the patient.^6,9^

## CASE REPORT

**Figure 1:**
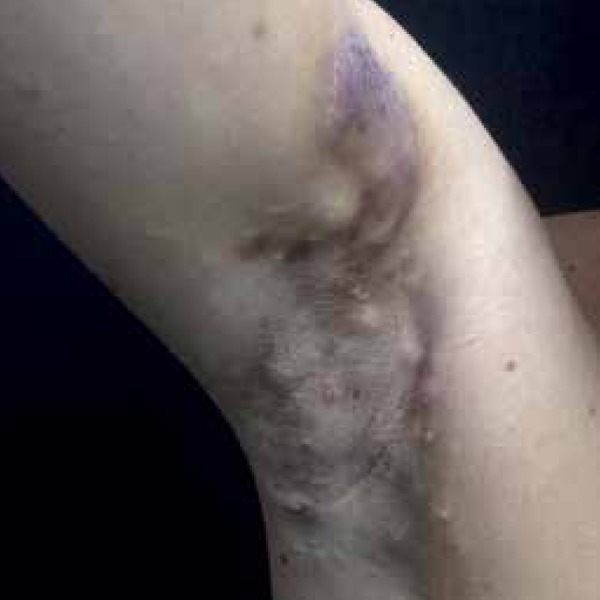
Nodulocystic lesions in the right armpit, res emb l in g hidradenitis

**Figure 2:**
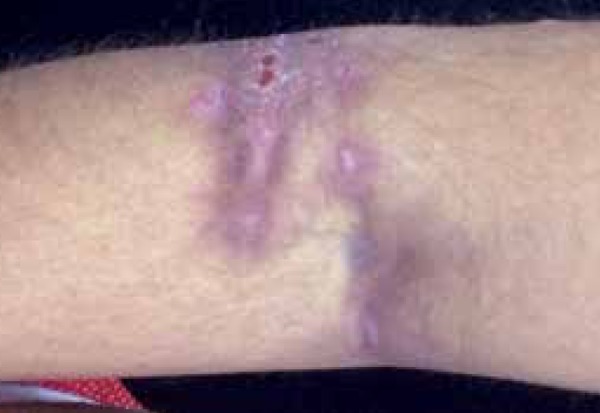
Lesion res emb l in g hidradenitis in the flexural area (right antecubital fossa)

Female patient, 23 years old, reported that at 11 years she noticed the appearance of papules and nodules in the axillary regions (Figure 1) and then in the inguinal region, which spread to trunk, lower limbs, antecubital fossae, face and scalp (Figures 2^,^3 and 4). She presented inflammation and recurrent infection of the lesions and underwent antibiotic therapy in these episodes. At 19 years, she used oral isotretinoin at a dose of 30 mg/day for 12 months, with slight improvement. She had no other comorbidities. The patient stated that her mother, who died of heart problems, had similar lesions on the face. The investigation proceeded with biopsy of a lesion, demonstrating epithelial cyst with thin eosinophilic cuticle in the inner part and adjacent sebaceous gland, findings that, associated with clinical findings of lesions resembling hidradenitis in flexural areas, confirmed the hypothesis of SM suppurativa (Figure 5). The patient did not want a surgical approach fearing the scars. Oral isotretinoin 1 mg/kg was then reintroduced and the patient improved clinically, maintaining the stability of the condition after six months of follow-up.

**Figure 3:**
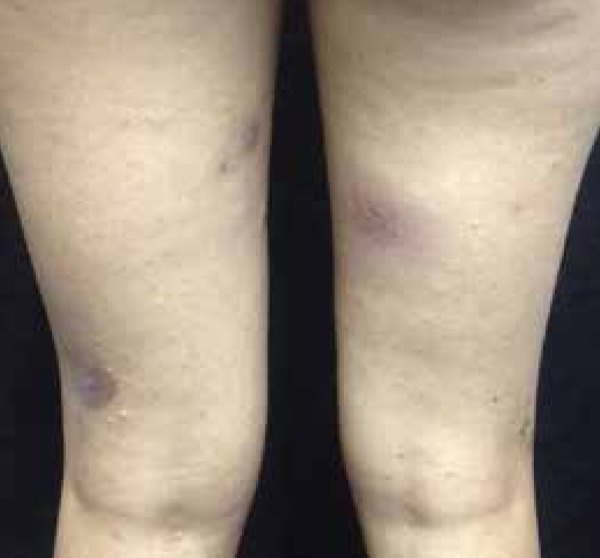
Residual hyperchromic lesions in the lower limbs

**Figure 4:**
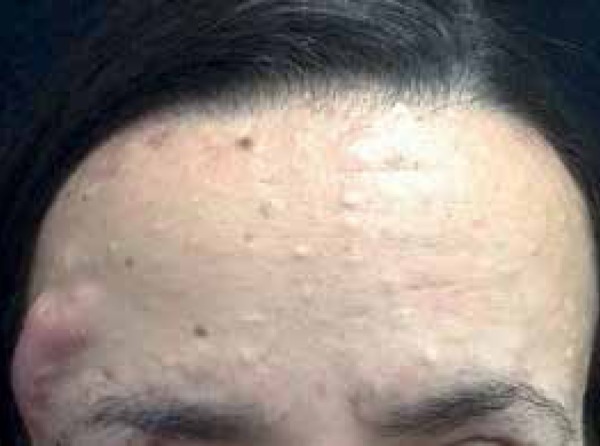
Papular and nodulocystic lesions on the forehead

**Figure 5:**
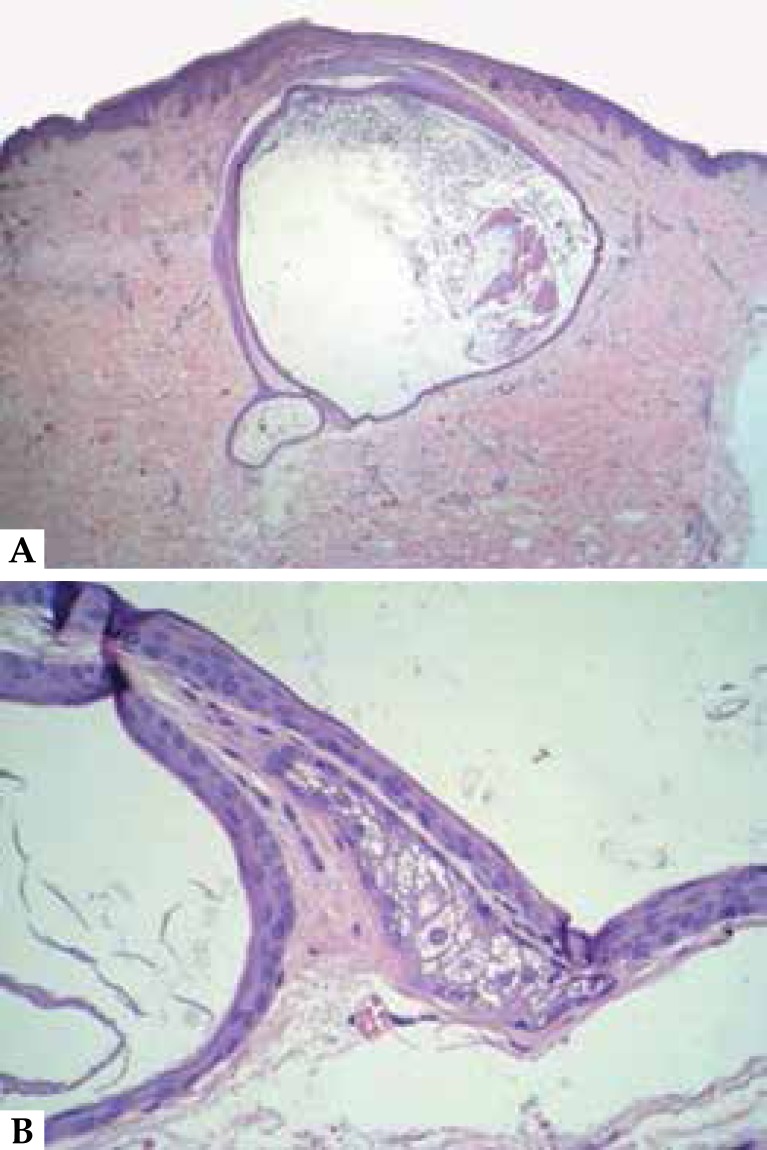
**A.** Epithelial wall cyst with thin eosinophilic cuticle in the inner part (HE 40X). **B.** Adjacent sebaceous gland is observed in the largest increase (HE 400X)

## DISCUSSION

SM is a hamartomatous malformation of the junction of the pilosebaceous duct.^1,7^ The origin of cyst formation is not completely known. The familial form may be related to mutation in the keratin 17, a keratin type 1 found in the sebaceous glands and hair follicles.^4^ The same mutation is found in pachyonychia congenita type 2 (PC-2), with which SM may be associated, a dominant autosomal disorder that presents with nail dystrophy, palmoplantar keratoderma, oral leukoplakia, follicular keratosis and epidermal inclusion cysts. ^2,4,5,8^ In addition, SM can manifest with hypertrophic lichen planus, acrokeratosis verruciformis, natal teeth and other findings.^5,8^

There are phenotypic variations within the same family, as in the case of our patient, and there may be overlap among subtypes. The same mutation in the keratin 17 gene may manifest with SM or PC-2 alone or in association. More than 11 distinct mutations are described; however, the resulting phenotype is independent of the type of mutation. It is possible that these manifestations are spectra of the same disease.^8,10^

The largest case series of SM comprises 64 patients with clinical, demographic and histopathology analysis. It has been demonstrated that most of the cases were sporadic, with mean age of 26 years, and the most affected sites were the arms (35%) and chest (29%).^3^

Symptoms such as pain, itching and fever may occur in the suppurative form. The evolution of SM to the suppurative form is rare and can happen at any time during the course of the disease. Reports of serious infections can result due to low socioeconomic status and poor adherence to treatment. Cystic lesions are often movable, with consistency ranging from firm to elastic. Nail disorder, such as hypertrophy, occur in most cases,^6^ but they were absent in the present case.

In addition to SM suppurativa, there are other reports of rare variants of SM, such as the facial, acral, vulvar and steatocystoma simplex (single lesion).^1,6^

In the case reported, although no genetic evaluation was performed, the patient probably had mutation in the keratin 17, considering it had a positive family history. Furthermore, it is a rare case, because it presented suppuration in limbs associated with facial and scalp lesions.

The clinic features of SM can resemble a number of diseases: vellus cysts, myxoid cyst, milia, acne conglobata, hidradenitis, and pseudofolliculitis, delaying the diagnosis and the proper monitoring.^1,2,4^ SM suppurativa must be distinguished from severe nodulocystic acne, acne conglobata, infected fibroadenoma, and pyoderma.^6^

To avoid delay in diagnosis of this condition, it is more feasible to proceed the investigation with histopathology,^1^ being more accessible than the genetic evaluation, in addition to presenting peculiar characteristics.

Histologically, the steatocystomas are dermal cysts coated with typical stratified squamous epithelium, whose stratum corneum shows wavy appearance, refractive and strongly eosinophilic. Sebaceous glands are usually present in the cyst wall, and hair may occur in its cavity.^1,2^

The treatment is varied and, in general, unsatisfactory, because of the difficulty of approaching such disseminated lesions. Surgery, needle aspiration, use of lasers such as CO_2_ and Erbium YAG, cryotherapy or oral isotretinoin are potential therapies options.^2,4,5^

Inflamed lesions can be addressed with injection of corticosteroids or drainage.^6^ Use of oral isotretinoin for the treatment of SM has been reported with mixed results. Most responsive patients had SM suppurativa.^9^ Isotretinoin usually does not eradicate this condition, but decreases the size of suppurative lesions.^6^ This therapeutic response probably reflects the anti-inflammatory effect of retinoids.^9^

These therapeutic modalities may be used in isolated form or in combination, according to the availability, clinical manifestations and patient’s wish, since most patients seek dermatologic service for esthetic demands.^2^ We should be alert to SM suppurativa and all the social and psychological implications it entails, seeking diagnosing as early as possible, clarifying the benign nature of the lesions, and offering available therapeutics.
